# Body mapping of sweating patterns of pre-pubertal children during intermittent exercise in a warm environment

**DOI:** 10.1007/s00421-021-04811-4

**Published:** 2021-09-21

**Authors:** Leigh Arlegui, James W. Smallcombe, Damien Fournet, Keith Tolfrey, George Havenith

**Affiliations:** 1 Environmental Ergonomics Research Centre, Loughborough School of Design and Creative Arts, Loughborough University, Loughborough, LE11 3TU UK; 2 Decathlon SportsLab, 59650 Villeneuve-d’Ascq, France; 3 School of Sport, Exercise and Health Sciences, Loughborough University, Loughborough, LE11 3TU UK

**Keywords:** Sweat, Exercise, Sweat mapping, Thermoregulation, Heat, Paediatric

## Abstract

**Purpose:**

To determine sweating responses of pre-pubertal children during intermittent exercise in a warm environment and create whole-body maps of regional sweat rate (RSRs) distribution across the body.

**Methods:**

Thirteen pre-pubertal children; six girls and seven boys (8.1 ± 0.8 years) took part. Sweat was collected using the technical absorbent method in the last 5 min of a 30-min intermittent exercise protocol performed at 30 ℃, 40% relative humidity and 2 m·s^−1^ frontal wind.

**Results:**

Mean gross sweat loss (GSL) was 126 ± 47 g·m^−2^·h^−1^ and metabolic heat production was 278 ± 50 W·m^2^. The lower anterior torso area had the lowest RSR with a median (IQR) sweat rate (SR) of 40 (32) g·m^−2^·h^−1^. The highest was the forehead with a median SR of 255 (163) g·m^−2^·h^−1^. Normalised sweat maps (the ratio of each region’s SR to the mean SR for all measured pad regions) showed girls displayed lower ratio values at the anterior and posterior torso, and higher ratios at the hands, feet and forehead compared to boys. Absolute SRs were similar at hands and feet, but girls sweated less in most other areas, even after correction for metabolic rate.

**Conclusion:**

Pre-pubertal children have different RSRs across the body, also showing sex differences in sweat distribution. Distributions differ from adults. Hands and feet RSR remain stable, but SR across other body areas increase with maturation. These data can increase specificity of models of human thermoregulation, improve the measurement accuracy of child-sized thermal manikins, and aid companies during product design and communication.

**Supplementary Information:**

The online version contains supplementary material available at 10.1007/s00421-021-04811-4.

## Introduction

Thermoregulation is a biological control system designed to maintain body temperature within a safe range. Warm environmental conditions and/or moderate-to-high exercise intensities can disrupt the body’s heat balance and cause a rise in body temperature. During exercise in cool, neutral, or even mild heat environments, ambient temperature (*T*_a_) is lower than skin temperature (*T*_sk_). In these conditions children have been shown to effectively thermoregulate mainly via dry heat loss mechanisms, where convective and radiant heat exchanges occur between the skin and the surrounding air (Bar-Or 1980; Naughton and Carlson 2008). Children’s larger body surface area to mass ratio (BSA/M) compared to adults allows them to have effective dry heat losses (Davies 1981). This, along with their rapid vasodilatory responses, allows them to maintain effective peripheral blood flow to the skin to facilitate dry heat loss (Drinkwater et al. 1977). However, when *T*_a_ approaches or is greater than *T*_sk_ dry heat loss mechanisms are no longer effective and may even result in possible heat gain. Evaporative heat loss then becomes the main, or only, route of heat dissipation. It was initially thought that, in these cases, children were at a thermoregulatory disadvantage due to their lower whole-body sweat production and their lower single sweat gland output compared to adults (Drinkwater et al. 1977; Bar-Or 1980). However, it may be the case that children simply require less evaporative cooling. As they have a lower body mass, they would generate less metabolic heat during weight bearing exercises compared to adults (Havenith 2001). This has previously been reported in a study by Smith and Havenith (2012) who showed that males had a higher metabolic heat production during two 30-min bouts of continuous exercise at 60% and 75% *V*O_2max_ due to greater absolute workloads and higher body mass compared to the females. Males therefore had higher absolute gross sweat loss and regional sweat rates. Furthermore, more recent investigations suggest that children exhibit a higher sweating efficiency (Falk and Dotan 2011; Leites et al. 2016) due to them having smaller but clustered sweat droplets over a larger BSA/M ratio of skin (Inbar et al. 2004; Rowland 2008). This would, therefore, allow children to thermoregulate effectively despite their suggested less mature thermoregulatory development. This fine line between dry and evaporative heat loss mechanisms makes it difficult to pin-point whether children are at a real disadvantage in extreme hot environments, or they simply have different thermoregulatory strategies compared to adults. This study aims to investigate this further by studying different aspects of children’s sweating responses during exercise in a hot environment, as outlined below.

Whilst some evidence points towards disparate sweating responses between adults and children, there is a paucity of data relating to the sweat production of children during exercise. In particular, the local sweat rates at different body regions are poorly understood in paediatric populations. Studies have previously reported data on children’s whole body sweat rates (Drinkwater et al. 1977; Inbar et al. 1981, 2004; Meyer et al. 1992; Shibasaki et al. 1997a; Rivera-Brown et al. 2008; Leites et al. 2016), and only a few have investigated local sweat rates or sweat gland activity of a limited number of small, very localised areas of the skin during exercise protocols in different environmental conditions. This previous research has typically employed the use of ventilated sweat capsules, the starch-iodine or the macrophotographic techniques (Bar-Or 1980; Falk 1992; Shibasaki et al. 1997a, b; Inbar et al. 2004; Inoue et al. 2009). These methods provide useful information on sweat gland responses of a localised area of the skin. However, practically, while not impossible (Machado-Moreira et al. 2008), it is challenging to study local sweat rates throughout multiple locations (i.e. > 5), and more importantly over larger skin surfaces around the body simultaneously using this method, particularly during exercise.

A more comprehensive assessment of local sweat rates can be achieved using the technical absorbent material method to create whole-body sweat maps. This technique is used to quantify regional sweat rates across the majority of the body’s skin surface using technical absorbent material (Havenith et al. 2008; Smith 2009; Morris et al. 2013). Previous work using this technique has shown differences between men and women during different exercise intensities (Havenith et al. 2008; Smith and Havenith 2011, 2012, 2019), and differences between young and older adults (Coull et al. 2020). Aging has been shown to dictate thermoregulatory processes. This has been seen in older populations who display lower gross sweat loss and different body sweat distribution compared to younger adults generating the same metabolic heat production during exercise (Coull et al. 2020). These known differences between younger and older adults raise the question of whether there are also regional sweat rate differences across the body in children, and whether these are different to adults.

It has been suggested that there could be meaningful differences in sweating responses throughout different skin sites in children, however this is based on sweat capsule data of less than five sites (Shibasaki et al. 1997b; Inoue et al. 2009). Additionally, due to similarities in biological maturation at a pre-pubescent stage, it is suggested that both sexes would have similar sweating responses up to puberty (Falk 1998). However, to date, these findings cannot be corroborated with the currently available data on children’s local, or regional, sweat rates as these data only represent small areas of the skin in a small number of locations. The technical absorbent material method would provide the data on whole-body regional sweat rates and distribution, however this methodology has, to our knowledge, never been attempted with a paediatric population.

Therefore, the aim of this study was to determine the sweating responses of pre-pubertal children during mild exercise induced hyperthermia to create a whole-body map of their RSRs and distribution across the body. This was achieved by following an adapted version of the technical absorbent material method and exercise protocol tested on young and older adults. Comparing sweat maps of the boys and girls will allow a tentative analysis of sex differences in this pre-pubertal population.

## Materials and methods

### Participants

Thirteen healthy pre-pubescent children (seven boys, six girls) volunteered to take part in this study. Participant physical and physiological characteristics are shown in Table 1. This study was approved by the University Ethics Committee #R18-P119. Participants and their legal guardians were fully informed of all the experimental procedures before completing a health screen questionnaire and provided written assent and written informed consent, respectively. All participants were pre-pubescent and did not report any cardiorespiratory, endocrine or neuromuscular conditions. Stage 1 of biological maturity was confirmed by the child with the assistance of a parent via a five-point self-assessment of secondary sexual characteristics (Tanner 1962). On average participants scored 3.4 ± 0.4 on The Physical Activity Questionnaire for Older Children (PAQ-C) (0 being inactive, 5 being very active) (Kowalski et al. 2004).

**Table 1:** Participant characteristics for age, mass, stature, body fat %, body surface area, estimated $\dot{V}{\text{O}}_{{{\text{2peak}}}} ,$ mean whole-body gross sweat losses, metabolic rates over all trials, PAQ-C scores, and treadmill speeds

	Boys (*n* = 7)	Girls (*n* = 6)	All (*n* = 13)	Range	*p* values
Age (years)	8.1 (1.1)	8.0 (0.0)	8.1 (0.8)	7.0–10.0	0.736
Body mass (kg)	26.4 (1.7)	29.8 (3.9)	28.0 (3.3)	22.9–33.4	0.056
Stature (cm)	134.0 (6.0)	134.3 (4.6)	134.2 (5.2)	122.6–141.0	0.914
Body fat (%)	16.2 (2.4)	21.2 (2.9)	18.5 (3.6)	11.8–25.0	0.006*
Body surface area (m^2^)	0.98 (0.05)	1.05 (0.09)	1.01 (0.07)	0.89–1.12	0.104
Estimated $\dot{V}{\text{O}}_{{{\text{2peak}}}}$ (L·min^−1^)	1.70 (0.26)	1.63 (0.30)	1.67 (0.27)	1.30–2.13	0.625
Whole-body gross sweat loss (g·m^−2^·h^−1^)	145 (35)	104 (51)	126 (47)	31–214	0.032*
Whole-body GSL from pads (g·m^−2^·h^−1^)	117 (33)	78 (36)	99 (39)	15–190	0.005*
Metabolic rate (W·m^−2^)	302 (44)	250 (42)	278 (50)	187–359	0.054
PAQ-C (score)	3.5 (0.4)	3.3 (0.2)	3.4 (0.4)	3.0–4.2	–
Walking speed (km·h^−1^)	3.4 (0.9)	3.0 (0.3)	3.2 (0.7)	1.8–4.3	–
Running speed (km·h^−1^)	7.7 (0.8)	6.5 (0.5)	7.2 (0.9)	5.8–9	–

Data presented as mean (SD). *p* values shown for sex comparisons. *p < 0.05.

*PAQ-C* Physical Activity Questionnaire for Older Children (Kowalski et al. 2004)

### Preliminary session

Anthropometry including body mass (Metter Toledo kcc150 weighing scale, Leicester, UK) and stature (standard stadiometer) were taken. Body composition assessment was completed to estimate percentage body fat (Tanita Body Composition Analyser MC-780MA scale; Tokyo, Japan). Body surface area was estimated using the equation proposed for children by Haycock et al. (1978):

1 $${\text{BSA}} = \left( {H^{0.3964} \times W^{0.5378} } \right) \times 0.024265\left[ {\text{m}}^{2} \right],$$

Regional body dimensions were then measured to individualise the size and dimensions of each absorbent pad (Smith and Havenith 2011). Participants performed a submaximal incremental treadmill test to determine treadmill speed, heart rate and oxygen uptake ($\dot{V}{\text{O}}_{2}$) steady-state relationships. Participants completed a 5 × 3-min stage protocol starting at a speed of 3 km·h^−1^ and increasing 1 km·h^−1^ at each stage, until reaching 80% maximum heart rate (HR_max_). HR_max_ was calculated using 220-age (Riebe et al. 2018) as per design in the indirect calorimetry system. A 1% gradient was set throughout the full duration of the test and all tests were conducted at 21 ℃, 50% relative humidity (RH). Expired air and heart rate were measured continuously using the Quark CPET COSMED Pulmonary Function Equipment system (Rome, Italy), a paediatric exercise test mask and a heart rate chest strap synchronised to the COSMED system. Ratings of Perceived Exertion (RPE) were recorded at the end of each stage.

### Sweat pad preparation and application

The technical absorbent material method used previously with adults (Havenith et al. 2008; Smith and Havenith 2011) was adapted for use in children. This mainly consisted of using a lower number of pads per participant, setting warmer environmental conditions, and changing the exercise protocol to an intermittent exercise (as explained below). A set of 53 pads of absorbent material (product 2724, Technical Absorbents Ltd., Grimbsy, UK) were cut out for each participant according to their individual body measurements as seen in ESM1 (electronic supplementary material). Each individual pad was weighed in its corresponding labelled air-tight zipper bag and then placed with double sided tape on a custom-made plastic sheet designed to fit each participant’s tested body location. Prior to pad application participants performed a 25-min exercise protocol (described in the next section) on the treadmill to elicit a sweating response. Their skin was then dried with a towel before pad application. Pads were then applied to their corresponding area on the body and kept in close contact with the skin using stretch tight-fitting, easily removable long sleeve t-shirt or trousers (zips were incorporated along sides, arms and legs). After the 5-min sweat collection exercise period, each pad was removed, placed into its labelled air-tight zipper bag and re-weighed. Sweat collection of the hands was measured using appropriately sized 100% cotton gloves under tight-fitting latex gloves to prevent sweat evaporation. Application and removal of the pads in each session took approximately 1.5 min.

Material testing was performed on the technical absorbent material to determine its maximum absorbency capacity and drying rate characteristics. This was conducted to determine whether the pads would reach or come close to reaching their maximal absorption capacity, as well as to determine the magnitude of any sweat weight errors from drying during the period between pad removal from the skin and being bagged. These assessments were performed on the material and the gloves at 30 ℃, 40% relative humidity (the experimental climatic conditions) by applying a range of water moisture samples (set from the observed pad weights during the experiments) and measuring the drying rates (changes in weight) over 5 min. Maximum absorption capacity was also measured by fully immersing each material in water for 5 min and taking their weight pre- and post-immersion. Results indicated that pads that absorbed the most sweat (~ 0.55 g) during the experimental sessions used only 0.9% of the pad material’s maximum absorption capacity. The mean amount of sweat absorbed by the pads (~ 0.13 g) used 0.2% of maximum absorbency. The wettest glove (~ 1.02 g) used 52% of the glove material’s maximum absorption capacity, however the mean amount of sweat absorbed (0.68 g) used 35% of the glove material maximum absorption capacity. Drying rates showed a mean 0.18% loss of the absorbed moisture weight per minute of drying period, during which the pad samples were fully exposed to the environment (i.e. not bagged). So, in principle, an exposure of the pads to the air for up to 5 min would result in less than 1% error in the measurement. Therefore, it was confirmed that very minimal drying rate errors occurred during the experimental sessions in this study and no further corrections were necessary.

### Experimental protocol

**Fig. 1:**
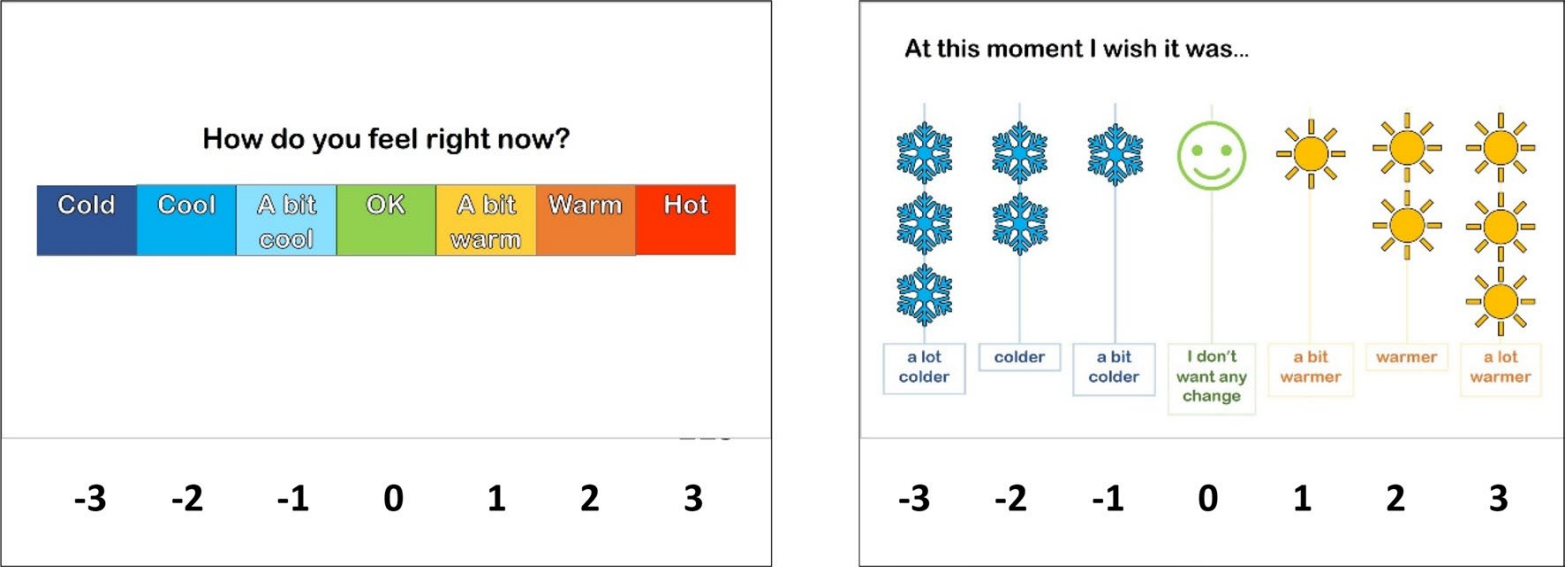
The thermal sensation and thermal preference scales used in the study adapted for children from Teli et al. (2012). Numerical equivalents are indicated below each point

Due to the high number of pads, and to avoid restricting sweat evaporation completely by covering too many body regions, sweat collection was done over three experimental sessions performed in an order counterbalanced between participants: session 1 (torso and feet), session 2 (arms and hands), and session 3 (legs and forehead). All sessions were conducted in a climatic chamber (30.3 ± 0.3 ℃, 40.8 ± 1.6% relative humidity) and 2 m·s^−1^ frontal wind speed. On arrival, participants changed into previously weighed short-sleeved t-shirt, shorts, socks, swimsuit top (for females) and shoes, and were asked to sit in a room at neutral temperature (~ 21℃). Resting heart rate (Polar A360 wristwatch) and tympanic temperature (Braun ThermoScan Pro 6000) were recorded at 3-min intervals for a total of a 15-min resting period. Tympanic temperature was measured solely as an ethical requirement for participant safety. Tympanic temperature was the chosen method of estimating body temperature due to ethical restrictions on the use of more invasive techniques, such as rectal sensors or ingestible telemetric pills. Participants rated their thermal sensation and thermal preference on adapted 7-point scales for paediatric use (Teli et al. 2012) (Fig. 1). Each descriptor equates to a numerical point on the scale (− 3 to + 3). “Cold” and “a lot colder” were − 3, “ok” and “I don’t want any change” were 0, and “Hot” and “a lot warmer” were + 3.

**Fig. 2:**
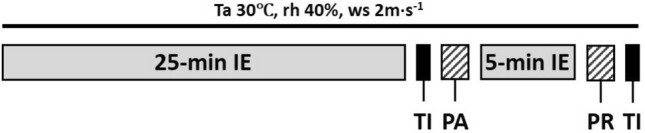
The exercise protocol in the climatic chamber. *Ta* ambient temperature, rh relative humidity, *ws* wind speed, *IE* intermittent exercise, *TI* thermal image, *PA* pads applied, *PR* pads removed

Participant’s body mass (Sartorius KCC150/ID7, Sartorius AG, Goettingen, Germany) was measured before entering the climatic chamber (TISS, Hampshire, UK) after which they completed a 25-min intermittent exercise on the treadmill. Intermittent exercise was chosen over continuous exercise (as previously employed on adults) based on the results of initial pilot tests of the experimental protocol. These revealed that pre-pubertal children were more engaged and motivated during an intermittent exercise and found the continuous exercise more challenging to complete. This consisted of alternating a 2-min walk at 45% HR_max_ with a 2-min run at 70% HR_max_. Given the long-time constant of the anticipated body temperature changes and the concomitant sweating responses, we assumed the walk/run protocol would still induce a steady state based on the average metabolic rate. Mean walking and running speeds were 3.2 ± 0.7 and 7.2 ± 0.8 km·h^−1^, respectively. Heart rate was recorded every min, and thermal sensation and thermal preference were recorded during the first min of each walking period. After completion of the 25-min exercise bout, whole-body thermal images (Flir T620 Infrared Camera, FLIR Systems Inc., Wilsonville, USA) of nude, dried skin were taken in shorts and swimsuit top (for females) before and after the absorption pad application to detect any changes in skin temperature during the sweat collection period. Skin was fully dried with a towel and pre-weighed pads were applied. Approximately 67% of the body (excluding groin, buttock, and main part of the head) was covered by absorption pads. The total area of the body covered by the pads for sessions 1, 2 and 3 were 0.20 ± 0.02 m^2^ (19.5%), 0.20 ± 0.01 m^2^ (19.6%) and 0.28 ± 0.03 m^2^ (28%), respectively. For females, the swimsuit top was also removed when applying the pads on the torso and back. After pad application, participants returned for a further 5-min of intermittent exercise on the treadmill (i.e. 2 min run, 2 min walk, and 1 min run), after which pads were removed immediately, placed into the individual air-tight zipper bags and weighed. The exact times were noted for each tested body section from which the pads were first in contact with the skin to when they were removed, giving exact application time in seconds (Fig. 2). Body mass was measured, and participants rested for another 15-min period where heart rate and tympanic temperature was measured every 3 min. End of trial ratings of thermal sensation and thermal preference were recorded and the clothing worn was weighed.

### Outcome variable calculations

The surface area of each pad was calculated by dividing the pad’s dry weight in grams by the weight of a square meter of dry absorbent material. The difference in weight before and after the sweat collection period gave the amount of sweat absorbed per pad in grams. Absolute RSRs (g·m^−2^·h^−1^) were then calculated by determining the grams of sweat absorbed per pad divided by its surface area and by pad application time. These data were used to create a sweat map of absolute regional sweat rates. To perform comparisons between child sweat distribution (rather than sweat rates) to other adult sweat maps, the sweat data were normalised per individual in relation to the sum of the area weighted sweat rates of all their measured pad regions. While both median (IQR) and means (SD) are tabulated, the median of each area of all participants was then taken to create the normalised sweat maps. Therefore, a ratio value of 1 for the local sweat rate is a median sweat rate equivalent to that for the whole body (based on the patch data), < 1 would be lower than the median, and > 1 would be higher than the median. These were calculated as described by Smith and Havenith (2019):

2 $${\text{RSR}}_{{{\text{norm}},i}} = \frac{{{\text{RSR}}_{i} }}{{\left\{ {\frac{{\mathop \sum \nolimits_{j = 1}^{j = n} \left( {{\text{area}}_{j} *{\text{RSR}}_{j } } \right)}}{{\mathop \sum \nolimits_{j = 1}^{j = n} \left( {{\text{area}}_{j} } \right)}}} \right\} }} ,$$

RSR_norm,i_ is the normalised local sweat rate of area *i* (dimensionless); RSR_i_ is the absolute regional sweat rate of area *i* (g·m^−2^·h^−1^); n is the number of tested areas; RSR_j_ is the absolute regional sweat rate of area *j* (g·m^−2^·h^−1^); area_j_ is the surface area of area *j*.

Gross sweat loss was calculated according to each participant’s body mass change before and after the exercise protocol. Corrections were applied to account for respiratory and metabolic mass losses:

3 $${\text{GSL}} = {\text{Body mass change}} - \left( {{\text{resp mass loss}} + {\text{met mass loss}}} \right)\left[ {{\text{g}} \cdot {\text{m}}^{-2} \cdot {\text{h}}^{ - 1} } \right],$$

where *resp* mass loss is respiratory mass loss; *met* mass loss is metabolic mass loss. Calculations for these are described below.

Mass loss from evaporative respiration (*E*_res_) and metabolic mass loss values were estimated as described previously by Smith & Havenith (2011):

4 $$E_{{{\text{res}}}} = 1.27 \times 10^{ - 3} \cdot M\left( {59.34 + 0.53 \cdot T_{a} - 11.69 \cdot P_{a} } \right) \left[ {{\text{W}}\cdot{\text{m}}^{ - 2} } \right],$$

where *M* is metabolic rate (W) (Eq. 8); *T*_*a*_ is ambient temperature (℃); *P*_*a*_ is the partial pressure of water vapour in the air (kPa).

5 $${\text{Respiratory mass loss }} = E_{{{\text{res}}}} \cdot t{ } \cdot { }\frac{1}{2430}\;\left[ {\text{g}} \right],$$

where *E*_res_ is evaporative loss from respiration (W); *t* is the duration of the experiment (seconds); and 2,430 is the latent heat of evaporation of 1 g of water (J·g^−1^).

6 $${\text{Metabolic mass loss}} = { }\left( {\frac{{\dot{V}O_{2} \left( {44 \cdot {\text{RQ}} - 32} \right)}}{22.4}} \right) \cdot t\left[ {\text{g}} \right],$$

where $\dot{V}{\text{O}}_{{2}}$ is oxygen consumption (L min^−1^); RQ is the respiratory quotient; and *t* is the duration of the experiment (min). Body mass, respiratory and metabolic mass losses were combined to give total mass loss in grams. This total mass loss (g) together with body surface area (m^2^) and the duration of the experiment (hour) gave GSL (g·m^−2^·h^−1^) values for each trial. As 3 trials were performed on different days, and the data are combined to produce a single body map, corrections were made to account for any day-to-day variation in GSL between trials (Smith and Havenith 2011, 2012). Additionally, GSL was also calculated from the total amount of sweat collected in the pads, where an overall whole-body GSL value was calculated:

7 $${\text{GSL}}_{{{\text{pads}}}} = \frac{{\mathop \sum \nolimits_{j = 1}^{j = n} \left( {{\text{area}}_{j} *{\text{RSR}}_{j } } \right)}}{{\mathop \sum \nolimits_{j = 1}^{j = n} \left( {{\text{area}}_{j} } \right)}}\left[ {{\text{g}} \cdot {\text{m}}^{-2} \cdot {\text{h}}^{ - 1} } \right],$$

The heat balance equation is a numerical representation of the body’s temperature regulation system. It shows how much heat is generated by the body (metabolic heat production), and the heat that is lost from the body to the environment (dry heat loss and evaporative heat loss). Metabolic heat production estimated as described by Weir (1949), where the oxygen consumption ($\dot{V}{\text{O}}_{{2}}$) values and respiratory exchange ratios (RER) for each stage of the intermittent exercise were taken from the submaximal exercise test data conducted during the preliminary session.

8 $$M = \dot{V}O_{2} \frac{{\left( {\left( {\frac{{{\text{RER}} - 0.7}}{0.3}} \right)e_{c} } \right) + \left( {\left( {\frac{{1.0 - {\text{RER}}}}{0.3}} \right)e_{f} } \right)}}{{\left( {60} \right)\left( {{\text{BSA}}} \right)}}\left( {1000} \right) \left[ {{\text{W}}\cdot{\text{m}}^{ - 2} } \right],$$

where *M* is metabolic rate (W); $\dot{V}{\text{O}}_{{2}}$ is oxygen consumption (l·min^−1^); RER is the respiratory exchange ratio; *e*_*c*_ is the energy equivalent for carbohydrates (21.13 kJ/l O_2_); *e*_*f*_ is the energy equivalent for fat (19.62 kJ/l O_2_); BSA is body surface area (m^2^).

Infrared thermal images were analysed using Flir ThermaCam Researcher Pro software. Changes in mean skin temperature values and standard deviations for forehead, torso, back, arms, hands, legs and feet pre- and post-pad application were determined using the analysis technique described in Quesada (2017; chapter 7). This consisted of pre-setting environmental, skin emissivity (0.98) and image set-up parameters (air temperature, humidity and distance from the participant to the camera) into the software. Images were then opened, and a shape selection was made by drawing around the edges of the targeted body area (e.g. hands) using a free-draw function. Analysis would then give the average, minimum, maximum and standard deviations of skin temperature within the selected shape. An infra-red black body calibrator (Omega BB702) was used in each picture to improve the absolute accuracy of the measurement.

Children’s sweat maps were assessed against the already existing adult sweat maps (Smith and Havenith 2011, 2012) to assess differences in sweat distribution. Boys and girls were assessed collectively to show the overall picture representing children. However, sweat maps were also made for girls and boys separately to determine if there are any differences in sweating responses between pre-pubescent boys and girls.

### Statistical analyses

Data were analysed using IBM SPSS Statistics version 25.0 (IBM Business Analytics, US). Descriptive data were presented as means with corresponding standard deviations or medians with interquartile ranges. Tests for normality were performed using the Shapiro–Wilk statistic, homogeneity of variance using Levene’s test of equality of error variances, and Mauchly’s Test for sphericity, where *p* values for significance were set to 0.05. Participant characteristics of age, body mass, stature, body fat and body surface area were analysed to detect any sex differences using independent samples *t* tests. Tests were run to confirm homogeneity across the 3 experimental sessions (session 1—torso and feet; session 2—arms and hands; session 3—legs and forehead). A one-way ANCOVA analysis was performed on the heart rate, using pre-test heart rate values as a covariate. Gross sweat loss and metabolic rate data were analysed using repeated measures ANOVAs. GSL, GSL calculated from the sweat content in the pads, and metabolic rate values were further analysed using 2 × 3 (sex by experimental session) mixed ANOVAs. GSL between sexes was also analysed with an ANCOVA, with metabolic rate as the covariate. Thermal sensation and preference data were treated as ordinal. A Quade’s rank analysis of covariance was used to assess if there were any differences in thermal sensation and thermal preference ratings between experimental sessions. Additionally, the data were processed to group all the sessions into one mean value for each time point throughout the experimental protocol (i.e. six time points from minute 4 to 24). Wilcoxon signed-rank tests were conducted with Bonferroni corrections adjusted for the number of comparisons made to assess the statistically significant differences between the scores at minute 4 to all the remaining time points. Median plane (left–right side) differences in absolute regional sweat rates were assessed using a 2 (median plane) by 24 (body sites) repeated measures factorial ANOVA. Pre- and post-pad application skin temperatures were analysed using a 2 (pre- and post-) by 19 (body sites) repeated measures factorial ANOVA. Independent samples *t* tests were performed to assess differences in regional absolute sweat rates and regional sweat rate ratio values between boys and girls (*p* value set to 0.05). Magnitude of effect sizes were set as proposed by Cohen (1988) to small (0.01), medium (0.06) and large (0.14) partial eta squares (*η*^2^_p_) values.

## Results

Participants displayed similar physical characteristics (Table 1). Participants walked at a mean speed of 3.2 ± 0.7 km·h^−1^ and ran at a mean speed of 7.2 ± 0.9 km·h^−1^, as per measured during their submaximal incremental treadmill test.

### Heart rate, GSL, metabolic rate and thermal perception

Heart rate, GSL and metabolic rate data for each session are presented in Table 2. Homogeneity was found in pre-test heart rate baseline values for walking and running heart rate analyses (*p* ≥ 0.751). ANCOVA analysis revealed no statistically significant differences between sessions for walking (*p* = 0.417, *η*^2^_p_ = 0.05) nor running (*p* = 0.473, *η*^2^_p_ = 0.04) heart rates. Repeated measures ANOVA analyses revealed no main effect between sessions for GSL (*p* = 0.906, *η*^2^_p_ = 0.01) nor metabolic rates (*p* = 0.264, *η*^2^_p_ = 0.11). Collectively, these results indicate that participants were exercising at similar intensities throughout each experimental trial as per experimental design. There were no statistical differences between sessions in thermal sensation (*F*(2,36) = 0.427, *p* = 0.656, *η*^2^_p_ = 0.02) and thermal preference ratings (*F*(2,36) = 0.03, *p* = 0.971, *η*^2^_p_ = 0.002).

**Table 2:** Mean (SD) exercise-induced physiological responses across the experimental sessions

Physiological variables	Session 1	Session 2	Session 3	Mean
Walking heart rate (b·min^−1^)	113 (8)	112 (8)	108 (11)	111 (7)
Running heart rate (b·min^−1^)	142 (9)	145 (6)	142 (6)	143 (5)
Whole-body gross sweat loss (g·m^−2^·h^−1^)	124 (44)	130 (47)	124 (53)	126 (47)
Metabolic rate (W·m^−2^)	273 (47)	284 (52)	277 (50)	278 (50)

Session 1—torso & feet; session 2—arms & hands; session 3—legs & forehead; heart rates are shown as the mean over the 25-min exercise protocol; whole-body gross sweat loss is the sweat loss value calculated from pre- & post-body mass measurements; metabolic rates are estimated values as described in Eq. (8). All comparisons across the sessions were non-significant (*p* = 0.264–0.804)

### Skin temperature

**Fig. 3:**
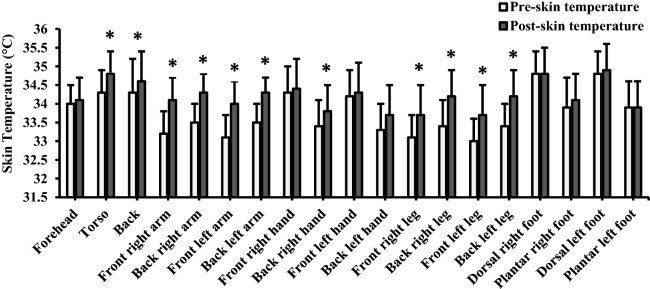
Mean (SD) pre- and post-skin temperature changes after the application of the absorbent pads. *Statistically significant differences pre- vs post-skin temperature (*p* ≤ 0.002)

Mean skin temperatures before and after the pad application were determined for forehead, torso, back, arms, hands, legs and feet (left and right sides, and front and back when applicable). There was a mean increase in skin temperature of 0.5 ± 0.5 ℃ across the body. When analysed as separate regions, there were statistically significant increases in skin temperatures between 0.3 and 0.9 ℃ in 11 out of 19 regions analysed (*p* ≤ 0.002) with the feet areas consistently unaffected (Fig. 3, ESM2 electronic supplementary material).

**Fig. 4:**
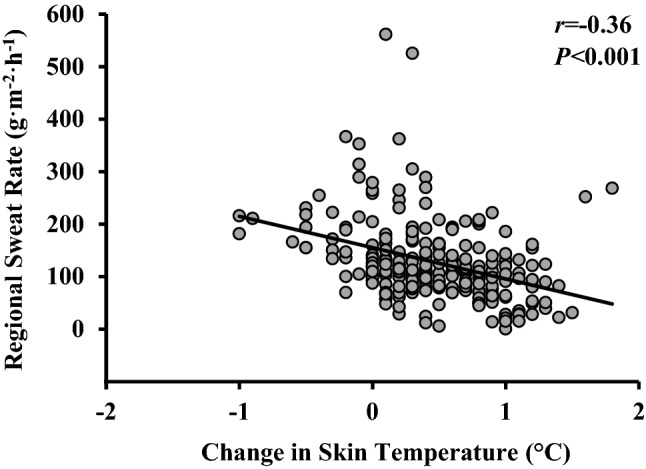
Relationship between regional sweat rates against changes in skin temperature for all 19 regions analysed

A Pearson’s analysis showed a significant but very weak, negative correlation between RSR and post- pad application skin temperature (*r* = − 0.19, *p* = 0.005), indicating that local skin temperature did not have a meaningful influence on RSRs. Similarly, a weak, but also negative relationship was found between RSRs and the change in skin temperatures over the pad application period (*r* = − 0.36, *p* < 0.001) (Fig. 4).

### Mean GSLs and metabolic rates

Table 1 shows the mean values of GSL (pre- and post-body mass), the total GSL calculated from the sweat content in the pads (GSL_pads_), and metabolic rate data from sessions 1, 2 and 3 combined. There were statistically significant differences between sexes in GSL (*p* = 0.032, *η*^2^_p_ = 0.35) and GSL from pads (*p* = 0.005, *η*^2^_p_ = 0.53). Differences in metabolic rate came close to significance with a large effect size (*p* = 0.054, *η*^2^_p_ = 0.30), suggesting a need for metabolic rate correction in the analysis of GSL. Boys exhibited higher values compared to the girls.

**Fig. 5:**
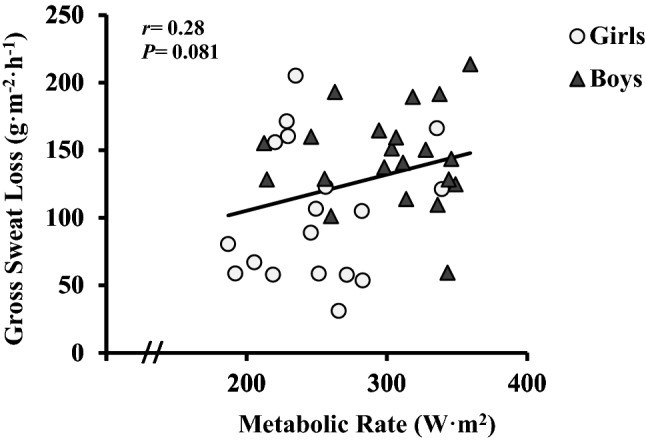
Relationship between whole-body gross sweat loss and metabolic rate. Boys are indicated by dark grey triangle; girls are indicated by light grey circle. Black trendline represents the full dataset of boys and girls

A Pearson’s correlation analysis across all data showed positive, weak correlations between GSL and metabolic rates (*r* = 0.28, *p* = 0.081) (Fig. 5). When boys’ and girls’ whole-body GSL were compared with metabolic rate as the covariate (ANCOVA), the sex difference, though reduced, remained significant (*p* = 0.028, *η*^2^_p_ = 0.127).

### Regional sweat rates (RSRs)

**Fig. 6:**
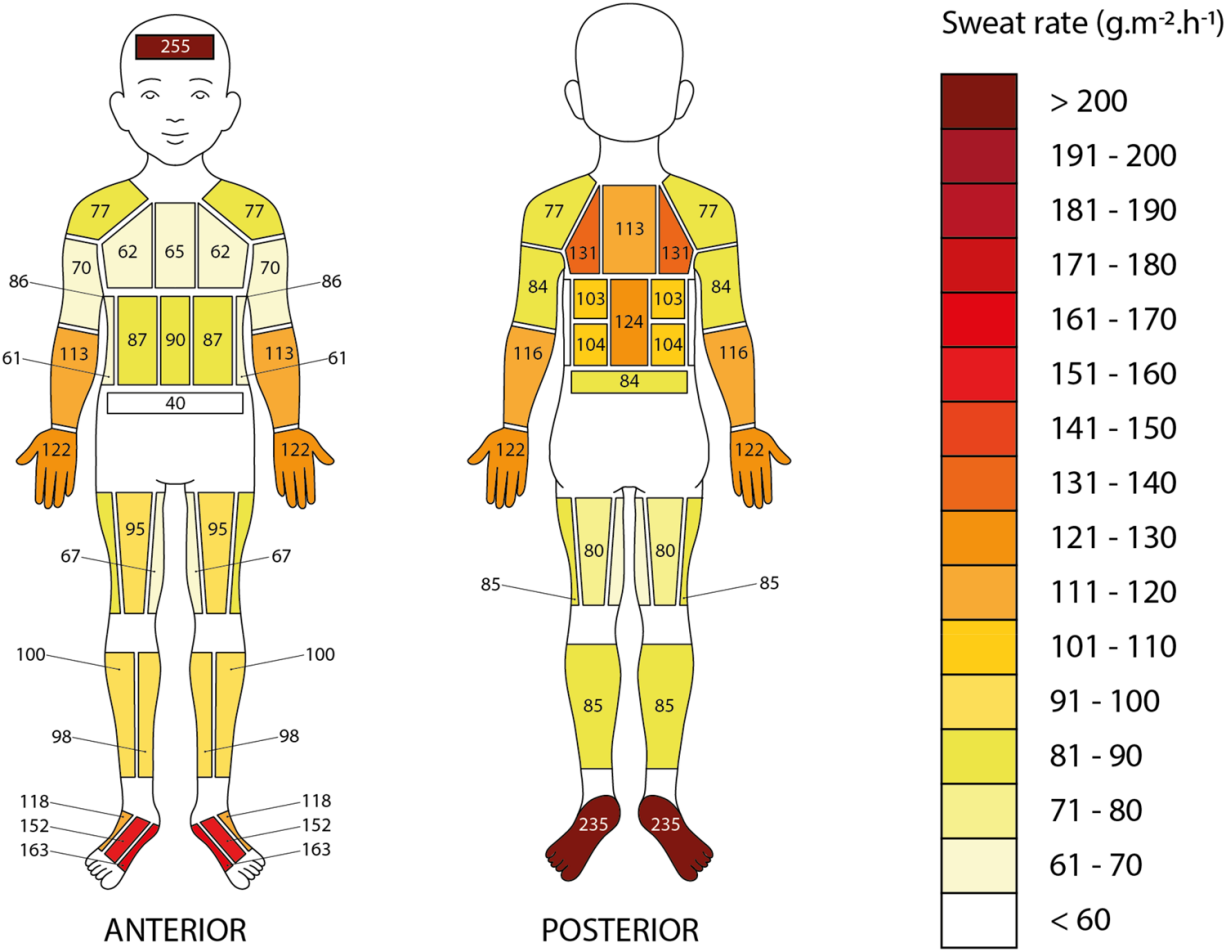
Absolute median regional sweat rates (g·m^−2^·h^−1^) of 13 pre-pubertal children after a moderate intensity treadmill exercise in the heat (note: the colour gradient scale was adjusted specifically for this dataset, hence it is different to earlier published maps for adults)

**Fig. 7:**
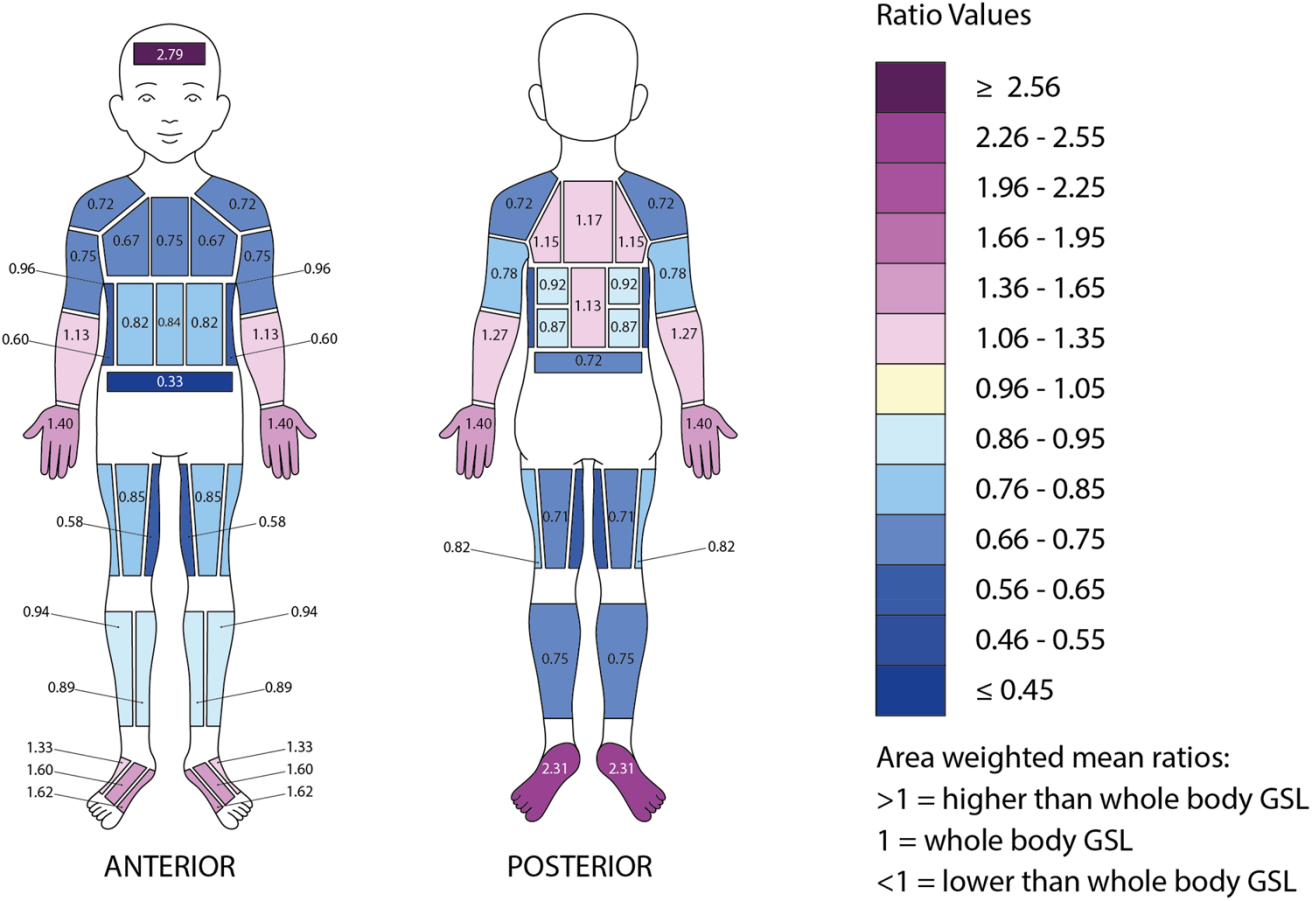
Normalised median regional sweat rates of pre-pubertal children after a moderate intensity treadmill exercise in the heat (1 = median sweat rate)

Means, medians and other summary statistics for all regional body zones are presented in Table 3. Maps showing whole group (n = 13) absolute RSRs and normalised RSRs can be seen in Figs. 6 and 7, respectively. The data in the sweat maps are presented as median values to show the “typical sweater” instead of showing a mean value as these would potentially have been influenced by very low or very high sweaters (Havenith et al. 2008). The median plane analysis indicated the differences in RSR between the left and right body sides were not significant (*p* = 0.449). Left and right mean values were therefore averaged for diagrammatic representation, as done previously in Havenith et al. (2008).

**Table 3:** Descriptive statistics of the surface areas of the pads, mean left–right absolute regional sweat rates (g·m^−2^·h^−1^), and normalised ratio data

Pad location	Area	Absolute sweat data (g·m^−2^·h^−1^)								Normalised ratio data	
	(cm^2^)	Mean	SD	Min	Max	Median	Q1	Q3	IQR	Median	IQR
Forehead	31	277	140	122	561	255	160	323	163	2.79	1.38
Shoulders	110	70	44	0	168	77	32	97	64	0.72	0.35
Lateral upper chest	69	66	45	0	165	62	38	97	60	0.67	0.41
Medial upper chest	76	75	53	4	174	65	49	115	66	0.75	0.49
Lateral mid anterior torso	78	74	48	0	161	87	46	104	59	0.82	0.43
Medial mid anterior torso	78	76	49	0	165	90	49	100	52	0.84	0.39
Sides	81	62	45	0	141	61	29	99	70	0.60	0.54
Lower anterior torso	77	40	36	0	108	40	19	51	32	0.33	0.23
Lateral posterior upper torso	83	108	57	1	202	131	53	143	89	1.15	0.37
Medial posterior upper torso	1	118	55	50	200	113	60	158	99	1.17	0.37
Lateral posterior mid upper	39	92	48	14	180	103	52	120	69	0.92	0.37
Lateral posterior mid lower	39	95	54	0	188	104	61	114	52	0.87	0.36
Centre posterior mid	80	111	57	13	207	124	71	145	75	1.13	0.46
Posterior lower torso	72	73	43	0	142	84	49	103	54	0.72	0.31
Armpits	49	108	87	0	312	86	51	146	95	0.96	0.72
Anterior upper arms	153	72	29	17	127	70	55	90	35	0.75	0.22
Posterior upper arms	151	74	30	11	115	84	58	96	38	0.78	0.14
Anterior lower arms	134	112	44	27	196	113	77	140	63	1.13	0.58
Posterior lower arms	135	123	47	58	215	116	84	157	73	1.27	0.59
Hands	417	135	39	73	208	122	106	158	53	1.40	0.46
Anterior upper legs	182	88	43	12	191	95	66	117	51	0.85	0.48
Medial upper legs	184	63	35	4	135	67	37	81	44	0.58	0.18
Posterior upper legs	182	76	39	7	151	80	51	96	45	0.71	0.39
Lateral upper legs	183	83	40	8	175	85	56	106	49	0.82	0.20
Lateral lower legs	145	93	42	10	174	100	64	121	57	0.94	0.24
Medial lower legs	145	99	43	20	176	98	82	124	42	0.89	0.52
Posterior lower legs	308	76	35	16	145	85	44	99	55	0.75	0.23
Centre dorsal foot	32	156	62	57	289	152	113	198	85	1.60	0.82
Medial dorsal foot	33	164	80	11	335	163	115	220	105	1.62	0.76
Lateral dorsal foot	33	131	70	38	310	118	92	157	65	1.33	0.58
Plantar foot	110	230	69	69	366	235	191	268	77	2.31	0.68

Absolute RSR data show higher sweat rates at the posterior torso compared to the anterior torso, similarly to adults. The regions with the lowest sweat rates were found at the lower anterior torso, the sides of the torso, the medial upper chest and the medial upper leg area. The lowest value was the lower anterior torso area with a median (IQR) sweat rate of 40 (32) g·m^−2^·h^−1^. In contrast, the highest sweat rates were found at the forehead, the plantar area of the feet, the medial and central dorsal foot areas, and lateral posterior upper torso area. The highest was the forehead with a median sweat rate of 255 (163) g·m^−2^·h^−1^ (Fig. 6). The map showing normalised median regional sweat rates shows a similar pattern, where the area with the lowest sweat rate in relation to the whole-body mean (= 1) was the lower anterior torso area (0.33 ± 0.31 ratio) and the highest was the forehead (2.79 ± 1.31 ratio) (Fig. 7).

**Fig. 8:**
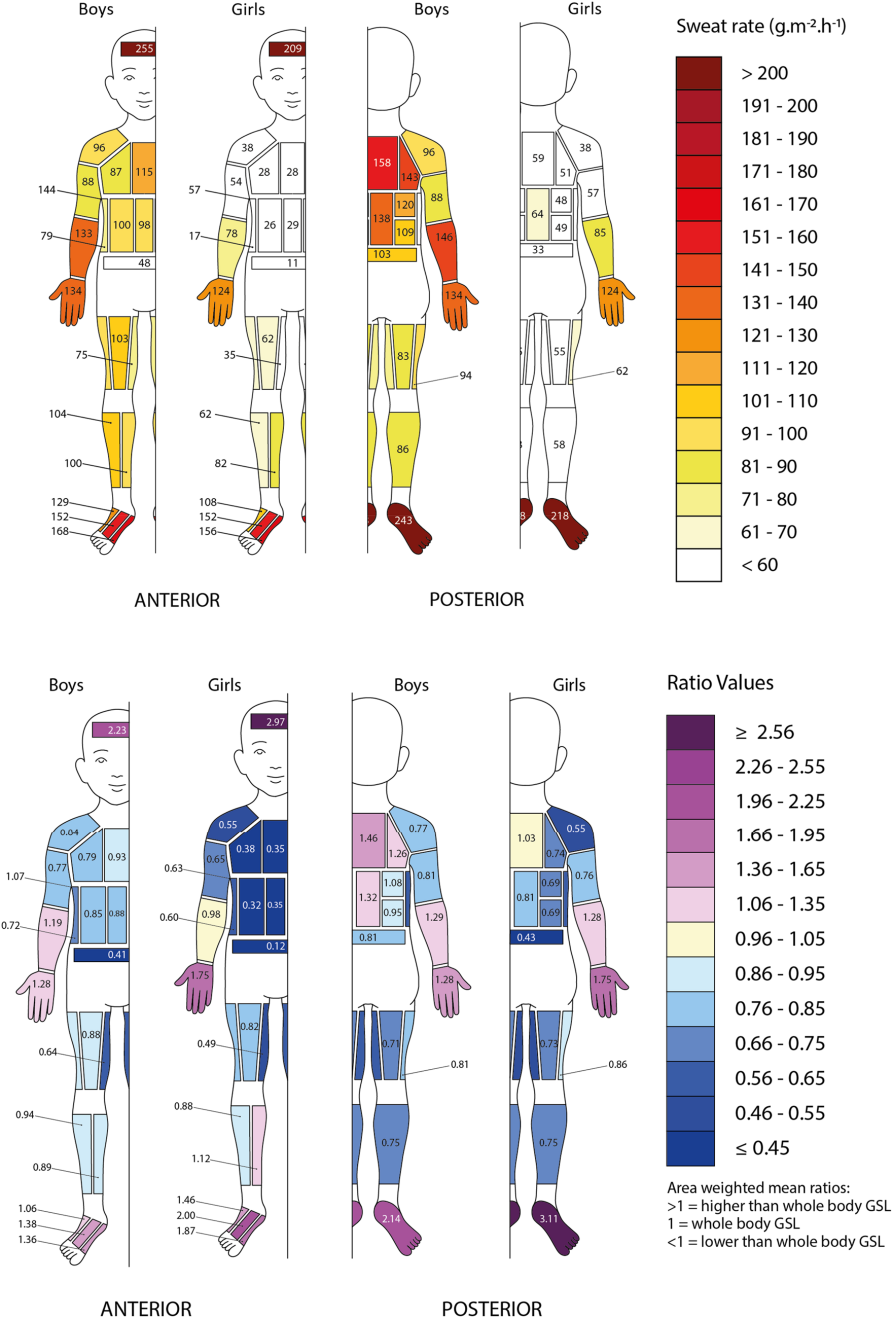
Absolute and normalised median regional sweat rates of boys and girls after a moderate intensity treadmill exercise in the heat (1 = median sweat rate)

Figure 8 shows the maps of boys and girls separately. Girls display lower ratio values at the anterior and posterior torso, and higher values at the hands, feet and forehead compared to the boys. This can be seen for example at the forehead (girls 2.97 vs boys 2.23), hands (girls 1.75 vs boys 1.28) and feet (plantar—girls 3.11 vs boys 2.14).

## Discussion

The main findings of this study show that pre-pubertal children do exhibit substantially different regional sweat rates across the body, as seen in the sweat maps. This provides clear evidence for the whole-body surface area of differences observed earlier in small area sweat capsule studies (Shibasaki et al. 1997b; Inoue et al. 2009). Additionally, there are also indications of sex differences in sweat rate distribution. Furthermore, as discussed below, the sweat distribution maps differ considerably from those of adults. Strikingly, sweat rates of hands and feet resemble each other between sexes and between ages, with substantial differences between sexes and ages observed for the rest of the body.

### Whole group between-session heart rates, GSL, thermal perception and skin temperatures

There were no individual differences in measures of heart rate, GSL (Table 2) and thermal sensation and thermal preference scores between sessions indicating that participants worked at similar exercise intensities during each of their three repeated experimental trials as intended per the experimental design. Skin temperature was measured primarily to determine the influence of the exercise protocol and the pad application procedure on skin temperatures. This was carried out to check whether the methodology elicited skin temperature increases that could have impacted RSRs. Results showed that on average skin temperatures changed between 0.1 and 0.9 ℃ during pad application, where the highest mean skin temperature measured was 34.9 ℃. These are similar skin temperature increases to those measured in male adults performing a run at a targeted heart rate of 150–160 b·min^−1^ for 60 min at 25 ℃, 53% relative humidity (mean temperature was 34.1 ℃) (Havenith et al. 2008). In line with the findings of previous studies (Smith and Havenith 2011, 2012), there was only a weak but negative relationship between RSRs and local *T*_sk_. These results indicate that these changes in skin temperatures, if any, should not have influenced RSRs.

### Whole group sweat maps of pre-pubertal children

Researchers have previously studied local sweat rates by determining sweat gland output and density in children using ventilated sweat capsules, starch-iodine or macrophotographic techniques. From these, it was suggested that there could be meaningful differences in sweating responses across different skin sites in children (Shibasaki et al. 1997b; Inoue et al. 2009). However, no whole-body or large area local sweat rate data existed to verify this. The sweat maps created in the present study have now made these data available, showing that there are indeed remarkable variations in local sweat rates throughout the body in pre-pubescent children. The sweat rate distribution pattern throughout the body also differs substantially from that in adults in similar exercise and environmental conditions (Smith & Havenith, 2011). In descending order, and starting with the highest value, children’s forehead, foot regions, hands, forearms and along the centre and upper areas of the spine had the highest sweat rates. In contrast, adults had the highest sweat rates on the forehead, along the centre and upper areas of the spine, and across the lower back area (Smith and Havenith 2011). Unlike children, adults typically exhibited low to moderate relative sweat rates on their hands and feet relative to the rest of the body. This reversal of high relative sweat rates in hands and feet in children to low relative sweat rates in adults may be the most striking change in the sweat distribution pattern. Moreover, it was observed that absolute sweat rates at hands and feet were very similar in the boys and girls, while absolute hand and foot sweat rates are also very similar between the young adult and children experiments. Thus, it seems that it is the sweat rate increase for the rest of the body that is responsible for the relative distribution changes with age and for male versus female, and this supports the different sweat control patterns (activation pathways) for hands and feet versus the rest of the body suggested already in the seminal work of Kuno (1956).

Sweat rates at the forehead are high in both children and adults possibly due to sweat gland densities not changing significantly in this region with skin growth, compared with other areas which experience more extensive skin growth (Szabo 1967). It would therefore be expected that the body regions, which do experience greater skin growth during development would have lower sweat rates per square meter of skin due to reducing sweat gland densities. However, this is not the case as it has been seen that sweat rates at the “*T*” area of the upper back and along the spine are relatively high in children and then become one of the dominant sites for sweat production in adults. These regions of higher sweat rates may therefore be governed by sudomotor sensitivity and sweat gland output, and not necessarily by sweat gland density (Smith and Havenith 2011). Shibasaki et al. (1997b) investigated sweat gland output at the forearm, thigh, chest and back areas while participants sat in at room temperature with their legs submerged in a hot bath at 42 ℃. They reported mean sweat outputs per gland in the different regions in boys (3.02, 3.18, 3.88 and 4.82 µg·HASG·min^−1^ respectively) and in men (3.99, 5.78, 7.53 and 8.02 µg·HASG·min^−1^ respectively). These results showed that children did exhibit slightly higher sweat rates at the back compared to the other body sites tested; however, these differences were much more pronounced in the adult men.

### Boys vs girls sweat maps

Consistent with the higher GSL in the boys versus the girls, and despite the relatively small samples of girls and boys, the results showed that 26 out of the 55 tested regional sites had statistically significant higher absolute RSRs in the boys (*p* < 0.001–0.05). While this may be attributed in part to metabolic rate differences (*p* = 0.054), using metabolic rate as covariant still showed a lower GSL for the girls. The normalised regional sweat rate ratios also revealed statistically significant differences in 16 out of the 55 tested sites (*p* = 0.001–0.05). Given the high number of zones comparisons, the chance of finding a few spurious significant differences increases. However, the chance of randomly finding more than 16 out of 55 significant zonal differences at *p* < 0.05 is less than 7*10^–9^. Bonferroni or alternative corrections would on the other hand be too conservative (Perneger, 1998; Lloyd et al., 2015). When observing the maps showing the distribution of sweat across the body (Fig. 8), boys seem to exhibit different sweating patterns compared to the girls. Girls showed a more pronounced distribution towards the extremities whereas the boys displayed a more even distribution across the body. In both cases there were body regions with higher and lower sweat rates, however, the girls had a greater difference between the highest and the lowest ratio compared to the boys (girls 3.11 at the plantar feet vs 0.12 at the lower anterior torso; boys 2.23 at the forehead vs 0.41 at the lower anterior torso). Thus, while future studies may be able to define this further, there is clear evidence in our data that there are regional sweat rate distribution differences between boys and girls, and, given that hand and feet absolute sweat rates are similar, that girls sweat substantially less than boys across the rest of the body (Table 4). These sex differences were also seen in the adult normalised sweat maps. Adult females displayed a higher distribution towards the hands and feet compared to adult males, who had a higher sweat distribution towards the torso area (Smith and Havenith 2012). Why this sex difference is already present pre-pubertal, where it is typically assumed that biological sex differences are not really developed yet, needs further research. While part of this may be attributed to differences in fitness, body mass and composition and basically metabolic rate differences, our analyses showed that while the differences get smaller, they do remain when correcting the data for such factors. A limitation of the sex comparison is the small independent samples of boys and girls, which means we cannot be sure the result is representative of children per se and will need to be replicated with larger and more diverse samples. Nevertheless, the statistical significance points to a consistent difference in our sample, which we believe warrants inclusion to stimulate further research.

**Table 4:** Descriptive statistics of the surface areas of the pads, mean left–right absolute regional sweat rates (g·m^−2^·h^−1^), and normalised ratio data for girls and boys

Pad location	Girls (*n* = 6)						Boys (*n* = 7)						Female/male ratio
	Area	Absolute sweat data (g·m^−2^·h^−1^)					Area	Absolute sweat data (g·m^−2^·h^−1^)					
	(cm^2^)	Mean	SD	Min	Max	Median	(cm^2^)	Mean	SD	Min	Max	Median	
Forehead	30	250	155	122	525	209	31	300	133	160	561	255	0.83
Shoulders	121	41	32	0	84	38	100	95	38	32	168	96	0.43*
Lateral upper chest	72	43	50	0	130	28	66	86	31	46	165	87	0.50
Medial upper chest	80	43	49	4	127	28	72	102	42	49	174	115	0.42*
Lateral mid anterior torso	82	40	42	0	108	26	75	103	29	59	161	100	0.39*
Medial mid anterior torso	83	44	46	0	108	29	74	104	33	60	165	98	0.42*
Sides	84	28	34	0	105	17	78	90	33	43	141	79	0.31*
Lower anterior torso	80	25	42	0	108	11	74	53	26	19	97	48	0.47
Lateral posterior upper torso	86	53	24	1	100	51	81	155	25	128	202	143	0.34*
Medial posterior upper torso	95	69	23	50	106	59	89	160	34	113	200	158	0.43*
Lateral posterior mid upper	41	49	23	14	82	48	37	130	27	100	180	120	0.38*
Lateral posterior mid lower	40	62	59	0	173	49	37	122	30	93	188	109	0.51*
Centre posterior mid	83	68	47	13	145	64	77	148	34	117	207	138	0.46*
Posterior lower torso	74	35	29	0	76	33	70	105	19	84	142	103	0.33*
Armpits	50	55	41	0	130	57	48	154	91	13	312	144	0.36*
Anterior upper arms	164	52	19	17	85	54	144	90	24	49	127	88	0.58*
Posterior upper arms	162	57	34	11	101	57	141	89	17	56	115	88	0.64
Anterior lower arms	141	88	42	27	166	78	128	132	35	71	196	133	0.67*
Posterior lower arms	141	98	40	58	179	85	129	144	44	84	215	146	0.68
Hands	412	133	41	81	208	124	421	136	39	73	194	134	0.98
Anterior upper legs	200	68	48	12	136	62	167	106	31	66	191	103	0.64
Medial upper legs	200	44	34	4	97	35	169	80	27	40	135	75	0.55*
Posterior upper legs	201	58	39	7	116	55	166	91	33	40	151	83	0.64
Lateral upper legs	200	64	41	8	132	62	168	100	30	50	175	94	0.64
Lateral lower legs	147	71	51	10	174	62	142	111	20	80	150	104	0.64
Medial lower legs	148	80	50	20	161	82	142	114	30	86	176	100	0.70
Posterior lower legs	323	59	36	16	111	58	294	91	28	35	145	86	0.65
Centre dorsal foot	31	152	56	77	222	152	32	159	69	57	289	152	0.96
Medial dorsal foot	32	149	51	72	242	156	34	177	99	11	335	168	0.84
Lateral dorsal foot	32	110	41	38	168	108	33	150	85	40	310	129	0.73
Plantar foot	112	229	43	182	305	218	108	230	87	69	366	243	1.00

*Significant differences between sexes *p* ≤ 0.05

### Gross sweat loss

In this study, the exercise protocol performed in heat stress conditions 30 ℃, 40% RH, 2 m·s^−1^ air speed elicited a mean whole-body sweating response of 126 ± 47 g·m^−2^·h^−1^ at a mean metabolic rate of 278 ± 50 W·m^−2^. This value is lower than the results of other studies with pre-pubertal children possibly due to the nature of this study’s exercise protocol. Meyer et al. (1992) investigated the sweating responses of 9-year-old boys and girls during two 20-min bouts of cycling at 50% *V*O_2peak_ with 10 min of rest in 42 ℃, 18% RH. Sweat rates were approximately 180 g·m^−2^·h^−1^. However, in this case participants exercised at a higher ambient temperature and experienced a longer exposure to the heat, increasing the chances of heat storage and a rise in core temperatures. Leites et al. (2016) found sweat rates of 278 g·m^−2^·h^−1^ in 11-year-old boys performing four bouts of 20 min of cycling at a mean metabolic rate of 175 W·m^−2^ in 35 ℃, 35% RH. These participants elicited higher sweat rates despite them working at a lower metabolic rate compared to participants in this study. The participants in Leites et al. (2016) study were, however, already in pubertal stages of development, therefore, they may have had a more mature thermoregulatory system allowing them to have a stronger sweating response. Additionally, they also exercised in a slightly warmer ambient temperature for a longer duration. This may have allowed sweat rates to further increase towards their peak over time, which may explain the higher gross sweat loss values. Shibasaki et al. (1997a) managed to elicit a higher sweating response in 10-year-old boys cycling for 45 min at 40% *V*O_2peak_ in 30 ℃, 45% (241 g·m^−2^·h^−1^). Even though the conditions were similar to those of this study, the main difference again is that they exercised for a longer period of time hence allowing more time for the core temperature to rise and subsequently produce higher sweat rates. Inbar et al. (2004) also found higher sweat rates of 326 g·m^−2^·h^−1^, however, in this case Inbar et al. (2004) exposed 9-year-old boys to a substantially warmer and dryer environment (41 ℃, 21% RH, < 0.3 m·s^−1^). Caution should be taken when making these comparisons of absolute sweat rates between studies as they use different exercise protocols and climatic conditions. Different exercise protocols may cause different cardiorespiratory responses, and hence different metabolic heat production, even if participants work at similar relative intensities (Baquet 2017). Additionally, the shorter length of the exercise protocol in this current study may have limited the core temperature rise and thus not have allowed for sweat rates to reach their peak, whereas these previous studies performed longer protocols which may be the main explanation for their greater gross sweat loss values. In this regard, it would be more appropriate to compare relative sweat rate ratio values; that being an individual’s regional sweat rate in relation to their overall body’s average regional sweat rate.

Davies (1981) showed that pubertal children exercising in 21 ℃, < 50% RH and 2–4 m·s^−1^ had a 50/50 distribution of heat loss over dry and evaporative pathways, while adults in the same conditions lost approximately two thirds of their heat via evaporative mechanisms. These differences can be seen when comparing gross sweat loss to metabolic rate ratio data of children and adults performing in similar exercise intensities and climatic conditions (first 30-min data of Smith 2009; Smith and Havenith 2011, 2012). The sweat rate to metabolic rate ratio was 0.45 versus 0.55 (g·h^−1^·W) for the children versus adults. Thus, overall, the children produce less sweat per watt of metabolic rate than adults, hinting further at a lower contribution of evaporative heat loss for the pre-pubertal children. However, the lowest sweat rate to metabolic rate ratio was observed in adult females (0.37) who exhibited a marginally lower ratio than female children (0.42). In contrast, adult males (0.70) exhibited a higher ratio compared with their child counterparts (0.48). The observed ratio difference for children is therefore mainly caused by the difference between male adults and children.

Despite working at the same target heart rate exercise intensity, boys had significantly higher absolute GSL and higher metabolic rates (approaching significance) compared to the girls (Table 1). This is similar to the observation of Smith and Havenith (2011, 2012) when comparing sexes. The explanation is likely to be the same too. While the estimated *V*O_2peak_ was not significantly different, the lower treadmill speeds observed for the girls for the same submaximal heart rate as the boys points toward lower cardiorespiratory fitness levels compared to the boys. A higher cardiorespiratory fitness and concomitant higher work rate, and thus higher metabolic heat production for the same heart rate in boys, may, therefore, explain a good part of the sweat rate differences. However, as correcting for metabolic rate still resulted in a difference, it did not explain the sex difference completely. An additional contribution may be that the higher cardiorespiratory fitness is indicative of a better trained and effective thermoregulatory system, initiating thermoregulatory responses to heat sooner compared to the girls (Foster et al., 2020).

While differences in metabolic heat production in several studies explain an important part of the lower absolute sweat production observed in children compared with adults, the question still remains as to whether, in addition to lower sweat production, children actually have limited capabilities of sweat generation or whether they can maintain effective thermoregulation via dry heat loss mechanisms at warmer temperatures compared to adults who begin to rely mostly on evaporative heat loss. This may be further investigated by including exercise intensities which elicit a higher metabolic heat production, or more extreme climates to force a higher reliance on sweating. For studies of maximal sweat capacity, this would aim to reach a range closer to uncompensable heat stress, pushing sweat requirements closer to the individual’s limit, showing whether the child–adult discrepancy is consistent across heat loads. For studies of the DRY-EVAPORATIVE balance on the other hand, a range of mild climates with increasing reliance on sweating would be relevant to investigate.

## Conclusions

This study has shown that pre-pubertal children do have substantially differing regional sweat rates across the body. Their sweat rate distribution also differs from that observed in adults performing a similar exercise protocol to the children. The children exhibited higher relative sweat rates at the hands and feet compared to the adults who in turn had higher sweat rates at the back. As hand and foot sweat rates in this study were similar to our previous work with young adults, the distribution change with puberty is caused mainly by increasing sweat rates over the rest of the body. As expected, given the weight bearing exercise, the children had lower metabolic heat production and required lower whole-body gross sweat losses compared to adults exercising in similar conditions.

Despite the relatively small samples of girls and boys, there were clear statistically significant sex differences, with girls exhibiting more extreme sweat rate differences across the body. As hand and foot sweat rates were similar, this is driven by lower sweat rates in the girls across the rest of the body. The boys showed a more even distribution across the body. Boys had significantly higher GSL and RSRs, linked to their higher metabolic rates. Given that body weights were similar this must at least in part be attributed to them having a higher fitness level (and thus higher metabolic rate for a given heart rate) compared to the girls. Correction for metabolic rate did not, however, remove the sex effect. The data provided by the paediatric sweat maps generated can be used to increase the specificity and accuracy of models of human thermoregulation for a paediatric population, as well as improve the measurement accuracy and realism of child-sized thermal manikins. These data can also prove useful for companies to improve the design of and communication regarding sports clothing and equipment for children.

## Supplementary Information

Below is the link to the electronic supplementary material.

Supplementary file1 (DOCX 1907 KB)

## Data Availability

Data may be available upon request.

## Abbreviations

BSA/M: Body surface area to mass ratio
*E*_*res*_: Evaporative loss from respiration
GSL: Gross sweat loss
*HR*_*max*_: Maximum heart rate
RER: Respiratory exchange ratio
RPE: Rating of perceived exertion
RSR: Regional sweat rate
*T*_*a*_: Ambient temperature
*T*_*sk*_: Skin temperature
$$\dot{V}{\text{O}}_{{2}}$$: Oxygen uptake
