# Supplementary material for: Body mapping of sweating patterns of pre-pubertal children during intermittent exercise in a warm environment
Source: Eur J Appl Physiol. 2021 Sep 21;121(12):3561–76. doi: 10.1007/s00421-021-04811-4 (PMC8571233; doi:10.1007/s00421-021-04811-4)
Supplement: Supplementary file 1 — Supplementary file1 (DOCX 1907 KB) [file 421_2021_4811_MOESM1_ESM.docx]

**Electronic Supplementary Material**

1. Shoulders
2. Lateral upper chest
3. Medial upper chest
4. Lateral mid anterior torso
5. Medial mid anterior torso
6. Sides
7. Lower anterior torso
8. Lateral posterior upper torso
9. Medial posterior upper torso
10. Lateral posterior mid upper
11. Lateral posterior mid lower
12. Centre posterior mid
13. Posterior lower torso
14. Anterior upper leg
15. Medial upper leg
16. Posterior upper leg
17. Lateral upper leg
18. Lateral lower leg
19. Medial lower leg
20. Posterior lower leg
21. Anterior upper arm
22. Posterior upper arm
23. Anterior lower arm
24. Posterior lower arm
25. Hands
26. Centre dorsal foot
27. Medial dorsal foot
28. Lateral dorsal foot
29. Plantar foot
30. Forehead
31. Armpits


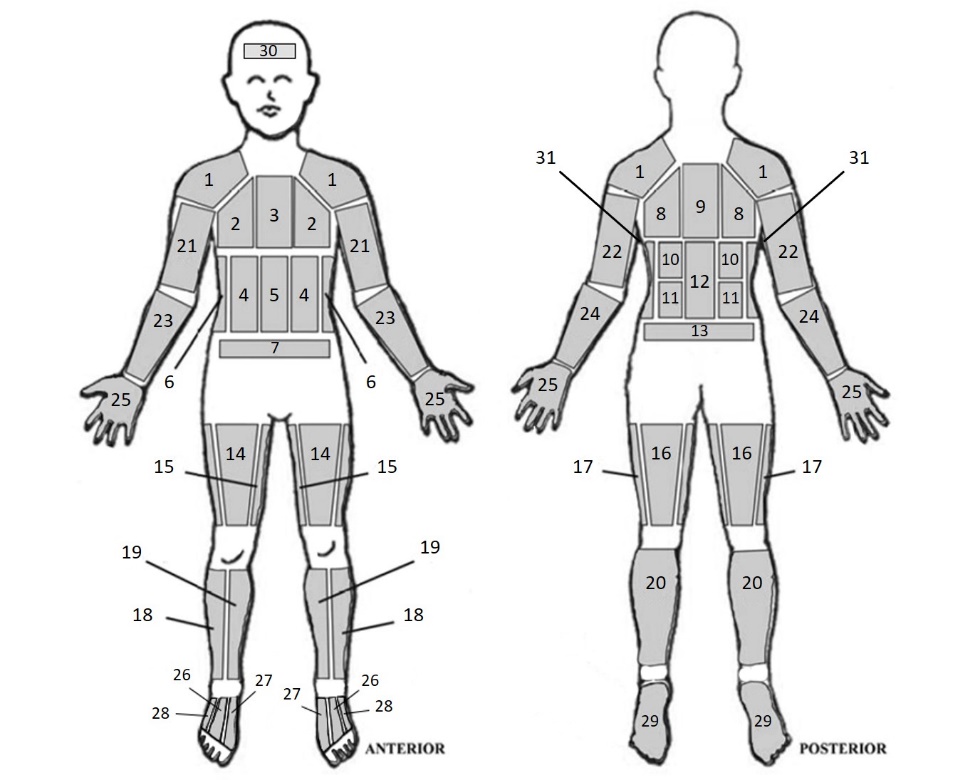


ESM1. The location and numeration of each absorbent pad on a participant (adapted from Smith, 2011). All the numbers correspond to a pad except for 25 which were cotton gloves. Numbers 31 are armpit pads.

ESM2. Mean ± SD pre- and post-skin temperature changes after the application of the absorbent pads. *Statistically significant changes from pre- to post-absorbent pad application *P≤* 0.05

|  | **Skin temperature (℃)** | | | | | |
| --- | --- | --- | --- | --- | --- | --- |
| **Location** | **Pre-** | **SD** | **Post-** | **SD** | **Change** | **SD** |
| Forehead | 34.0 | 0.5 | 34.1 | 0.6 | 0.1 | 0.3 |
| Torso | 34.3 | 0.6 | 34.8 | 0.6 | 0.5* | 0.2 |
| Back | 34.3 | 0.9 | 34.6 | 0.8 | 0.3* | 0.3 |
| Front right arm | 33.2 | 0.6 | 34.1 | 0.6 | 0.8* | 0.3 |
| Back right arm | 33.5 | 0.5 | 34.3 | 0.5 | 0.8* | 0.4 |
| Front left arm | 33.1 | 0.6 | 34.0 | 0.6 | 0.9* | 0.3 |
| Back left arm | 33.5 | 0.5 | 34.3 | 0.4 | 0.8* | 0.4 |
| Front right hand | 34.3 | 0.7 | 34.4 | 0.8 | 0.2 | 0.4 |
| Back right hand | 33.4 | 0.7 | 33.8 | 0.7 | 0.4* | 0.3 |
| Front left hand | 34.2 | 0.7 | 34.3 | 0.8 | 0.1 | 0.3 |
| Back left hand | 33.3 | 0.7 | 33.7 | 0.8 | 0.4 | 0.4 |
| Front right leg | 33.1 | 0.6 | 33.7 | 0.8 | 0.6* | 0.3 |
| Back right leg | 33.4 | 0.7 | 34.2 | 0.7 | 0.8* | 0.3 |
| Front left leg | 33.0 | 0.6 | 33.7 | 0.8 | 0.7* | 0.4 |
| Back left leg | 33.4 | 0.6 | 34.2 | 0.7 | 0.8* | 0.3 |
| Dorsal right foot | 34.8 | 0.6 | 34.8 | 0.7 | 0.0 | 0.4 |
| Plantar right foot | 33.9 | 0.8 | 34.1 | 0.7 | 0.2 | 0.7 |
| Dorsal left foot | 34.8 | 0.6 | 34.9 | 0.7 | 0.1 | 0.3 |
| Plantar left foot | 33.9 | 0.7 | 33.9 | 0.7 | 0.1 | 0.7 |
